# BLE Fingerprint Indoor Localization Algorithm Based on Eight-Neighborhood Template Matching

**DOI:** 10.3390/s19224859

**Published:** 2019-11-07

**Authors:** Mingfeng Li, Lichen Zhao, Ding Tan, Xiaozhe Tong

**Affiliations:** 1 School of Geomatics Science and Technology, Nanjing Tech University, Nanjing 211800, China; Jennnings@njtech.edu.cn (L.Z.); xztong0810@163.com (X.T.); 2 School of Geodesy and Geomatics, Wuhan University, Wuhan 430079, China; tanding-87@163.com

**Keywords:** indoor localization, fingerprint localization, eight-neighborhood, template matching, bluetooth low energy

## Abstract

Aiming at the problem of indoor environment, signal non-line-of-sight propagation and other factors affect the accuracy of indoor locating, an algorithm of indoor fingerprint localization based on the eight-neighborhood template is proposed. Based on the analysis of the signal strength of adjacent reference points in the fingerprint database, the methods for the eight-neighborhood template matching and generation were studied. In this study, the indoor environment was divided into four quadrants for each access point and the expected values of the received signal strength indication (RSSI) difference between the center points and their eight-neighborhoods in different quadrants were chosen as the generation parameters. Then different templates were generated for different access points, and the unknown point was located by the Euclidean distance for the correlation of RSSI between each template and its coverage area in the fingerprint database. With the spatial correlation of fingerprint data taken into account, the influence of abnormal fingerprint on locating accuracy is reduced. The experimental results show that the locating error is 1.0 m, which is about 0.2 m less than both K-nearest neighbor (KNN) and weighted K-nearest neighbor (WKNN) algorithms.

## 1. Introduction

Indoor localization has become more and more significant in location based service (LBS) [1,2]. Bluetooth [3,4,5,6], WIFI [7,8] and ultra-wide band (UWB) [9,10,11] are widely used in indoor localization systems based on received signal strength indication (RSSI) from the known access points (APs). Bluetooth low energy (BLE) has attracted increasing interests for its low-cost, low-power consumption and ubiquitous availability in mobile devices [12]. Similar with other wireless technologies, the BLE indoor localization based on RSSI can be divided into the trilateration algorithm and the method of location fingerprint [13]. In trilateration, the distances between the unknown points and APs are measured with acquired RSSI based on the signal propagation mode. Then the fingerprint localization can be divided into the offline phase and the online phase. During the offline phase, the RSSI distributions of different APs are determined by measuring the RSSI at different reference points (RPs), and a fingerprint database is generated. During the online phase, the unknown points are located with the best RPs, which are chosen based on the fingerprint database and the acquired RSSI from different APs. Due to the complex indoor environment, signal non-of-sight propagation and other factors, the RSSI does not always follow the signal propagation mode [14]. As a result, the accuracy of distance between the unknown point and the AP may not satisfy the requirement. In contrast, the fingerprint localization to distinguish the positions by the RSSI from different APs may be less affected by the environment. Therefore, the fingerprint localization may be more accurate than the trilateration algorithm, especially in a complex environment [15].

The best RPs which are the most important for the fingerprint localization, are usually determined with the K-nearest neighbor (KNN) algorithm, and the weighted K-nearest neighbor (WKNN) algorithm or their improved algorithms. Bahl et al. determined the best RPs by the KNN algorithm and located the unknown points with the error of 2–3 m [16]. Kukolj et al. matched the position fingerprint through signal simplification and principal component analysis to improve the WKNN algorithm [17]. Li Hualiang et al. applied the method of kernel function extraction to train the original fingerprint data, and matched the fingerprint data in the database with the error of 1.5 m based on an improved WKNN algorithm [18]. Bi Jingxue et al. considered the influence of the human body on the signal propagation and established an omnidirectional fingerprint database to locate the unknown points in WKNN algorithm [19]. The fingerprint localization requires an established RSSI database to store the coordinates and RSSI features from different APs of the RPs. For the low efficiency of the RSSI database establishment, Liu Huiyuan et al. proposed a new method based on an improved Kriging interpolation algorithm, and the experiments showed that the locating accuracy was kept and the workload was reduced by 50% [20].

In the methods mentioned above, k RPs with the most similar RSSI features to the unknown points are chosen. Because of different acquisition environments, signal strength is easily biased during RSSI acquisition [21], and it may happen both in the offline phase and the online phase which usually leads to unsuitable RPs. It is worth mentioning that if there are abnormal RSSI values in the original data, it is easy to influence the reliability of the peripheral interpolation points. Most fingerprint techniques are not satisfactorily performed due to the mismatch in offline training and online measurement caused by these abnormal RSSI values. To avoid the abnormal RSSI’s influence on the locating accuracy, we propose a method for fingerprint indoor localization based on eight-neighborhood template matching (ENTM), which generates eight-neighborhood templates with the RSSI values acquired at the unknown point, and the template matching is applied to choose the best RP as the estimation of location.

This paper is organized as follows: in Section 2, the algorithms of KNN and WKNN and the problems in application are introduced; the relationship of RSSI in fingerprint database is analyzed and the proposed ENTM algorithm is introduced in Section 3. In Section 4, the experiment and the analyzing results are presented; finally, the conclusions are presented in Section 5.

## 2. Related Works

### 2.1. Algorithms of KNN and WKNN

The KNN algorithm is one of the usual methods in data classification technology. First, Euclidean distances of RSSI vectors at the unknown point and the i-th RP in the fingerprint database are calculated as:

(1) $$D_{i}\;=\;\sqrt{\sum_{j=1}^{M}{\left(P_{r,\mathrm{dB}}\left(d^{j}\right)\;-\;P_{r,\mathrm{dB}}\left(d_{i}^{j}\right)\right)}^{2}},\;i\;=\;1,\;2,\;\cdots ,\;N,$$

where M is the number of APs in the indoor environment and N is the number of RPs in the fingerprint database. $P_{r,\mathrm{dB}}(d^{j})$ is the j-th AP’s RSSI acquired at the unknown points, and $P_{r,\mathrm{dB}}\left(d_{i}^{j}\right)$ is the RSSI of the same AP at the i-th RP stored in the fingerprint database. After calculating N distances, k RPs which has the smallest distances are selected to estimate the coordinates of the unknown point as:

(2) $$\left(x,\;y\right)\;=\;\frac{1}{k}\;\sum_{i=1}^{k}\left(x_{i},\;y_{i}\right),$$

where (x, y) are the estimated coordinates and $\left(x_{i},\;y_{i}\right)$ are the coordinates of the i-th RP.

WKNN is an improved algorithm of KNN and also selects k RPs to estimate the location of the unknown point. The impact of different selected RPs which have different distances from the RSSI vector at the unknown point, and the smaller distance indicates that the RP is closer to the unknown point than the others theoretically. To express the relevance, a different weighting coefficient is used for RPs in WKNN instead of the middle point of the selected RPs. The estimated coordinates are calculated as:

(3) $$\left(x,\;y\right)\;=\;\sum_{i=1}^{k}\;w_{i}\;\left(x_{i},\;y_{i}\right),$$

where $w_{i}$ is the weighting coefficient of the i-th RP, which can be determined by

(4) $$w_{i}\;=\;\frac{1/D_{i}}{\sum_{i=1}^{k}\;\left(1/D_{i}\right)},$$

where $D_{i}$ is the distance between the RSSI vectors of the unknown point and the i-th RP.

### 2.2. Problems in Application

**Figure 1:**
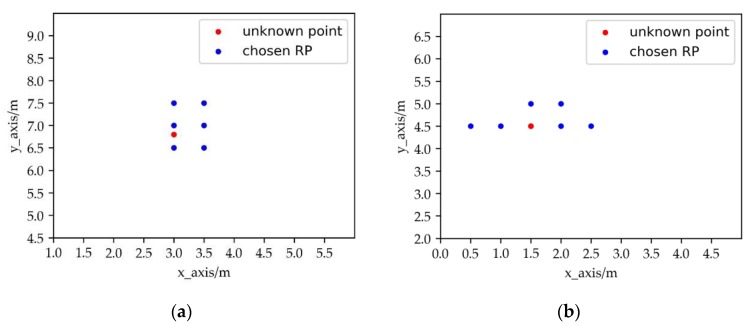
The normal distribution of reference points (RPs), where selected RPs gather around the unknown point in (**a**) and none of the selected RPs is significantly away from the cluster in (**b**).

The key basis for the algorithm to select the RPs is the similarity (e.g., Euclidean distance) between the RSSI vectors of RPs and the unknown point, so the fingerprint database is vital for the accuracy of the localization. As shown in Figure 1, the chosen RPs should cluster around the unknown point. However, it is shown that the signal propagation suffers from several noisy characteristics like temperature, the location of furniture, the multipath effect and even, the human body [22]. To solve the problem, there are many outlier suppression methods in indoor localization such as the median filter, the Kalman filter and RPCA (Robust Principle Component Analysis) [23]. In these methods, the single point reception RSSI value of the reference point is only taken into account, but there is still correlation between the feature RSSI values of the adjacent RPs. RPs which have abnormal feature RSSI values may still exist in the fingerprint database. For the algorithms of KNN and WKNN that select RPs by similarity, the abnormal RPs may affect the locating accuracy. It may be divided into two categories: some of the selected RPs are more similar to the unknown point but further from it and all the selected RPs do not gather around the unknown point. The cluster generated by the selected RPs is unstable from the perspective of cluster validation [24]. As is shown in the following figure, the number beside the selected RP represents the similarity order between the RSSI vectors of the RP and the unknown point.

**Figure 2:**
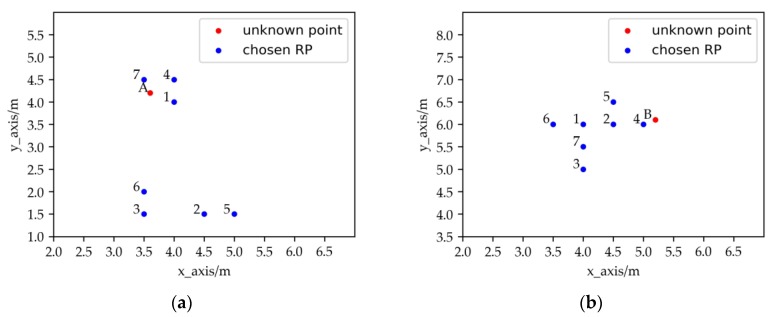
The abnormal distribution of RPs, where some of the selected RPs similar in RSSI to the unknown point is further in (**a**) and the selected RPs similar in RSSI to the unknown point gather on one side in (**b**).

In Figure 2a, Point 2 and Point 3 are more similar with the unknown Point A in the RSSI vector, but further from Point A. In Figure 2b, the chosen RPs do not cluster around Point B, and the middle point of the chosen RPs is far from Point B.

## 3. Proposed ENTM Algorithm

### 3.1. Template Matching and Eight-Neighborhood

**Figure 3:**
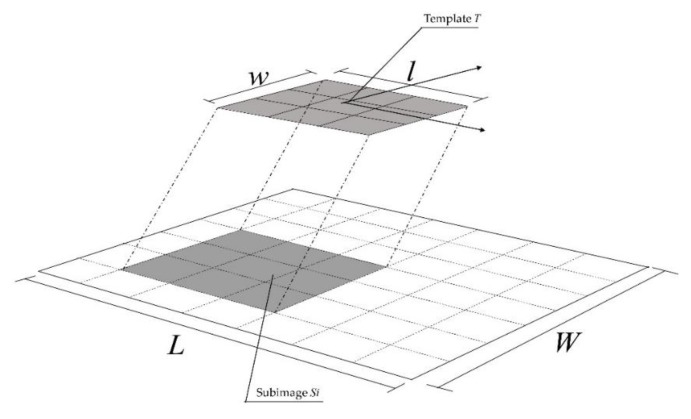
Schematic of template matching.

Template matching is an effective pattern recognition technology in image processing [25]. By calculating the correlation between template and region, the similarity between matching template and matching region may be more directly reflected. As shown in Figure 3, the template T is a known small image which has a size of l × w. The template matching is to search for a target in a large image which has a size of L × W (L > l, W > w), and the target is similar to the template in size and direction. During the matching process, T moves on the image, and the region where T is overlaid ($S_{i}$ in Figure 3) is called the subimage. The related coefficient between T and $S_{i}$ is calculated, and the subimage with the greatest correlation is considered as the target.

**Figure 4:**
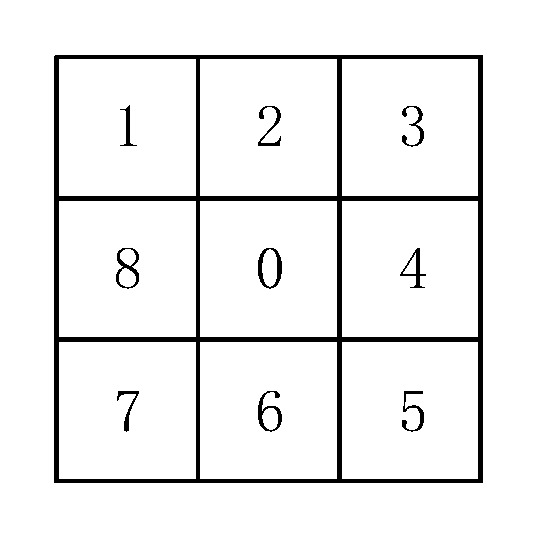
Schematic of the eight neighborhood.

Because the RSSI values of the adjacent RPs are highly correlated which will be explained in Section 3.2 and Section 3.3, the density of RPs may be increased by spatial interpolation and the distribution of the RPs may be converted into a regular grid form which is similar to raster image. Therefore, the method of template matching may be used to select the best RPs with the RSSI correlation taken into account. In order to reduce the influence of abnormal RPs and to take the correlation of signal strength, the 3 × 3 template or the eight-neighborhood template is selected as the matching template. As shown in Figure 4, the eight-neighborhood refers to the organization of data in a regular grid, which is centered on Grid No. 0 and neighboring Grid No. 1, 2 ⋯ 8 in all directions [26].

### 3.2. Spatial Resolution of BLE RSSI

The RSSI distribution in the AP is susceptible to the indoor environment, but still satisfies the general rule of signal propagation. Because the RSSI of the signal is continuously decreasing with the distance from the center AP, it is necessary to discuss the decrease of RSSI with the distance. BLE signal intensity attenuation model can be described as the log-normal distribution [27] which is:

(5) $$RSSI\;=\;RSSI_{d_{0}}-\;10n\log_{10}(\frac{d}{d_{0}})\;+\;X_{\sigma },$$

where $RSSI_{d_{0}}$ is the measured reference value at the distance of $d_{0}$, RSSI is the measured value at the distance of d, and n is the path loss exponent, which indicates the rate of increasing path loss related to the distance. $X_{\sigma }$ denotes a Gaussian random variable with zero mean caused by shadowing [28].

**Figure 5:**
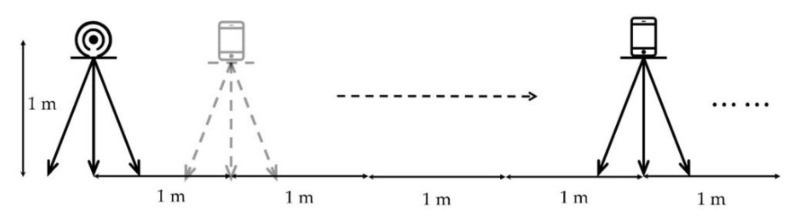
Schematic of data acquisition experiment.

As shown in Figure 5, an iBeacon is taken as a known AP and set at the certain height of 1 m from the ground, and the AP’s RSSI is acquired with a mobile phone at the horizontal intervals of 1 m at the same height in an open area. In the experiment, $d_{0}$ is chosen to be 1 m, $RSSI_{d_{0}}$ is −66.88. To simplify the calculation, $X_{\sigma }$ is set to be 0 due to the unencumbered experiment environment [29]. And the path loss exponent n is 1.263 which is determined by fitting with the acquired data.

Table 1 displays the RSSI values at the interval of 0.5 m, where a part of the RSSI values are obtained by fitting. The differences of RSSI values between each neighboring pair are also shown, which are calculated by:

(6) $$\Delta RSSI\;=\;RSSI_{i+1}\;-\;RSSI_{i},$$

where $RSSI_{i}$ is the estimated value of a certain distance and ΔRSSI is the difference at the neighboring distances.

It is found that the RSSI is weaker and the difference is more subtle with the increasing interval of distance. At the distance of 3.5 m, the difference of RSSI is less than 1 dBm, which means that the difference in RSSI values is little. Considering that the APs are normally placed at a higher position such as the ceiling, the RSSI values of adjacent RPs are close, especially when the RPs are densely distributed.

### 3.3. Correlation of Spatial RSSI Fingerprint

The indoor fingerprint database organized by the regular grid is similar to the raster image. Although there might be abnormal RPs, the RSSI values of the adjacent RPs are highly correlated in the most parts of the fingerprint database. The value of each grid in the eight-neighbor template indicates the RSSI of a certain AP signal that can be measured in the indoor environment, and the RSSI of the center grid is the measured RSSI data at the unknown point. Due to the fact that RSSI vectors of the eight neighboring grids cannot be obtained in a short time during the online phase, the RSSI data in the fingerprint database should be analyzed to find out the relationship between the central grid and its neighbors.

**Figure 6:**
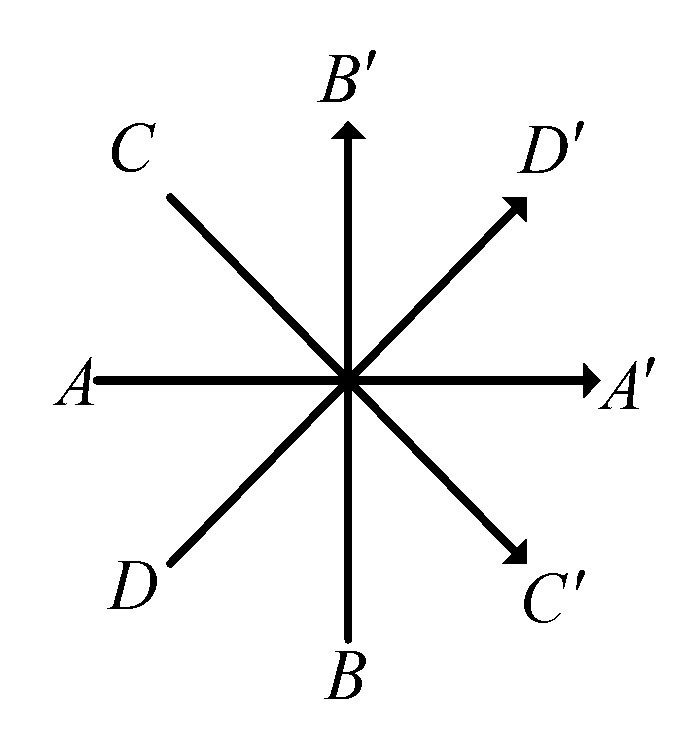
The four directions of the center point.

In order to discuss the distribution of the RSSI value of a single AP signal indoors, an AP is arranged at a certain point in the 8 m × 8 m room, and a separate coordinate system is established. The RPs are chosen at interval of 1 m, and the RSSI feature of each RP is determined by mean filtering. Considering the influence of the interpolation on the locating effect of the abnormal RP, the data is interpolated at an interval of 0.5 m by Kriging interpolation. As shown in Figure 6, the eight-neighborhood can be divided into four directions.

The differences between the RPs and its neighbor RPs in the four directions are calculated and recorded. Since the RSSI difference is described as a floating number, the results are processed as:

(7) $$\Delta R^{\prime }\;=\;floor\;(\Delta R\;+\;0.5),$$

where ΔR is the raw difference of the adjacent RPs in different directions, and $\Delta R^{\prime }$ is an integer (…,−3,−2,−1,0,1,2,3,…). The occurrences of each integer are recorded as n. In Figure 6, the histogram of RSSI differences in the direction of $AA^{\prime }$ is shown.

**Figure 7:**
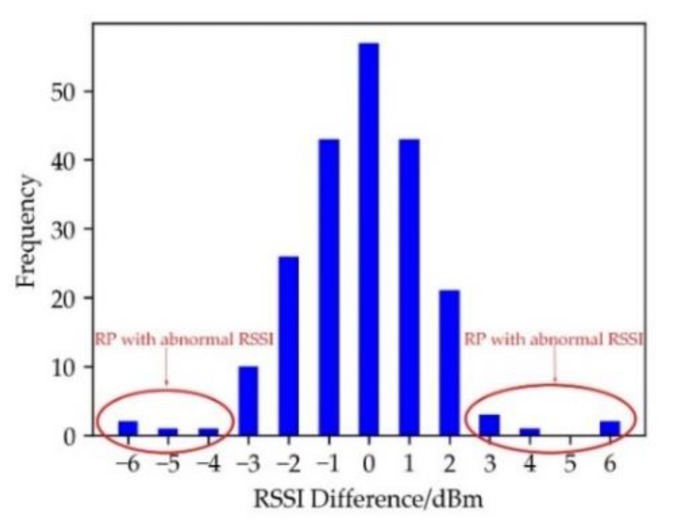
Histogram of RSSI difference in the direction of $AA^{\prime }$.

As shown in Figure 7, the RSSI difference of adjacent RPs is less than 3 dBm, which is consistent with the spatial resolution of RSSI. It is obviously that there exist still a few differences larger than 3 dBm, which may be regarded as the abnormal RPs. Therefore, the impact of abnormal RPs on the template generation parameters needs to be reduced during the generation of the eight-neighborhood template. Although the occurrences of RSSI differences throughout the indoor environment are similar to the normal distribution, the differences of RSSI between the center grid and its eight neighboring grids are related to both the position of the center grid and that of the AP. Since the RSSI value of the AP cannot be accurately collected during offline phase, the maximum position of the RSSI feature after the interpolation is selected instead of the AP position. With the selected point taken as the origin and the horizontal and vertical coordinates as the dividing lines, the indoor space can be divided into four quadrants. In Figure 4, the frequency of each RSSI difference in the fourth quadrant is shown.

**Figure 8:**
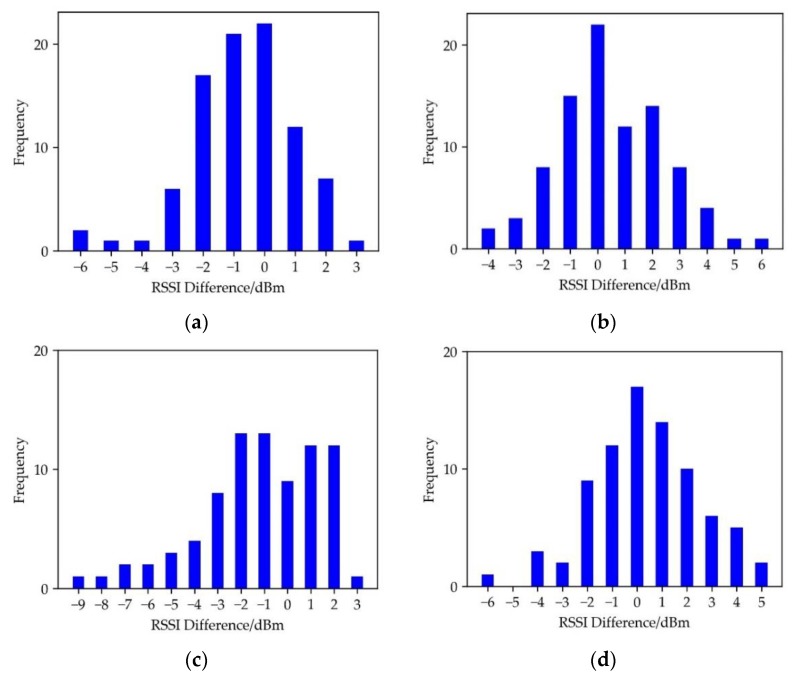
Histogram of ΔR in the fourth quadrant and (**a**) Histogram of ΔR in the direction of $AA^{\prime }$, (**b**) Histogram of ΔR in the direction of $BB^{\prime }$, (**c**) Histogram of ΔR in the direction of $CC^{\prime }$, (**d**) Histogram of ΔR in the direction of $D^{\prime }D$.

As shown in Figure 8, the RSSI difference of the neighborhood grid which is closer to the AP and the center grid is biased to the positive direction. Therefore, the quadrant and the location of the neighborhood grid should be taken into consideration to generate the template.

### 3.4. ENTM Algorithm

The proposed ENTM algorithm may be divided into the three steps, i.e., the generation of fingerprint database, the generation of the matching template and the location estimation.

(1) The generation of the fingerprint database

Assuming that there are *M* APs in the indoor environment, the RSSI of any point may be received as $\left(r_{1},\;r_{2},\;\ldots ,\;r_{M}\right)$. With the m-th AP taken as an example, the data is organized into a regular grid by interpolation to make the fingerprinting database similar to a raster image in structure. The RSSI distribution of the m-th AP may be written as:

(8) $$R_{m}=\;\left[\begin{array}{cccc} r_{\left(0,\;0\right)}^{m} & r_{\left(0,\;1\right)}^{m} & \cdots & r_{\left(0,\;V\right)}^{m}\\ r_{\left(1,\;0\right)}^{m} & r_{\left(1,\;1\right)}^{m} & \cdots & r_{\left(1,\;V\right)}^{m}\\ \vdots & \vdots & r_{\left(u,\;v\right)}^{m} & \vdots \\ r_{\left(U,\;0\right)}^{m} & r_{\left(U,\;1\right)}^{m} & \cdots & r_{\left(U,\;V\right)}^{m} \end{array}\right],$$

where $R_{m}$ is the RSSI distribution of the m-th AP, u (0 ≤ u ≤ U) and v (0 ≤ v≤ V) are the indices of the fingerprint, U and V are the maximum values of the indices which are determined by interpolation intervals of different axes, and $r_{\left(u,v\right)}^{m}$ is the feature RSSI of the RP determined by interpolation. With the interpolation intervals set, the coordinates of each RP may be described by:

(9) $$\{\;\begin{array}{c} x_{u}\;=\;x_{0}+\;u\Delta x\\ y_{v}\;=\;y_{0}+\;v\Delta y \end{array},$$

where Δx and Δy are the intervals of the grid, and $\left(x_{0},\;y_{0}\right)$ is the coordinate of the starting point in the grid.

(2) The generation of the matching template

The generation of eight neighborhood matching template needs to reduce the influence of the abnormal reference point and to follow the AP signal propagation law. Therefore, different matching templates need to be generated according to the position of the template center point. The expected values of the RSSI differences in different directions of each quadrant are used as the parameters to generate the template. The expected values may be determined by:

(10) $$E_{q}(h)\;=\;\sum \;\Delta R\;•\;p\left(\Delta R\right),$$

where ΔR is the RSSI differences processed from Equation (7), p(ΔR) is the probability of occurrence of ΔR, q is the number of quadrant, and h is the position of the neighborhood.

The RSSI value of each neighborhood point may be expressed as:

(11) $$r_{q}^{h}\;=\;r_{o}\;+\;E_{q}(h),$$

where $r_{0}$ is the RSSI vector acquired at the unknown point. The eight-neighborhood template in the q-th quadrant may be described as:

(12) $$T_{q}=\;\left[\begin{array}{ccc} r_{q}^{1} & r_{q}^{2} & r_{q}^{3}\\ r_{q}^{8} & r_{o} & r_{q}^{4}\\ r_{q}^{7} & r_{q}^{6} & r_{q}^{5} \end{array}\right].$$

(3) The location estimation

**Figure 9:**
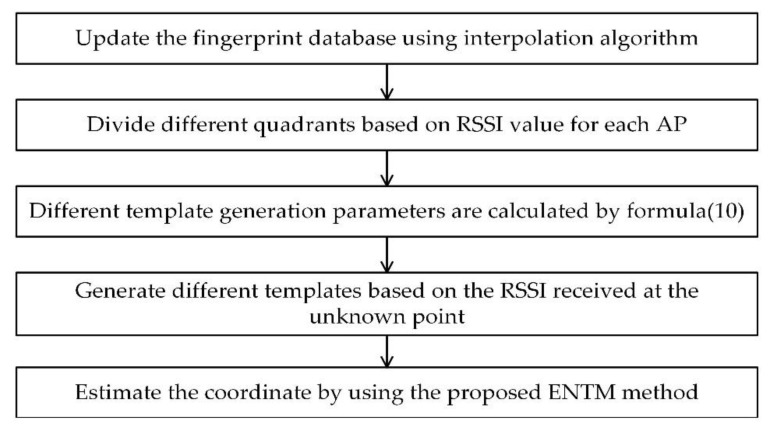
The flow chart of fingerprint indoor localization method based on the proposed eight-neighborhood template matching (ENTM) method.

In the location estimation, the matching template $T_{q}^{m}$ of the m-th AP is moved on $R_{m}$, and the quadrant q where the template is located is determined by the direction from the m-th AP to the center point of the template. The coverage area of $R_{m}$ is $S^{m}$, and the center point of $S^{m}$ is indexed with (u,v) in the fingerprint database. The similarity D between template T and sub-area S is calculated by Euclidean distance as:

(13) $$D\left(u,v\right)\;=\;\sum_{m=1}^{M}\;\sqrt{\sum_{\phi =0}^{3}\;\sum_{\varphi =0}^{3}{\left(S^{m}[\phi ][\varphi ]\;-\;T_{q}^{m}[\phi ][\varphi ]\right)}^{2}},$$

where ϕ and φ are the element in matrix $S^{m}$ and $T_{q}^{m}$. Therefore, the position of the RP with the minimum D is chosen as the estimated position. The flowchart of the proposed ENTM is shown in Figure 9.

## 4. Experiment and Analysis of Its Result

### 4.1. The Environment of Experiment

**Figure 10:**
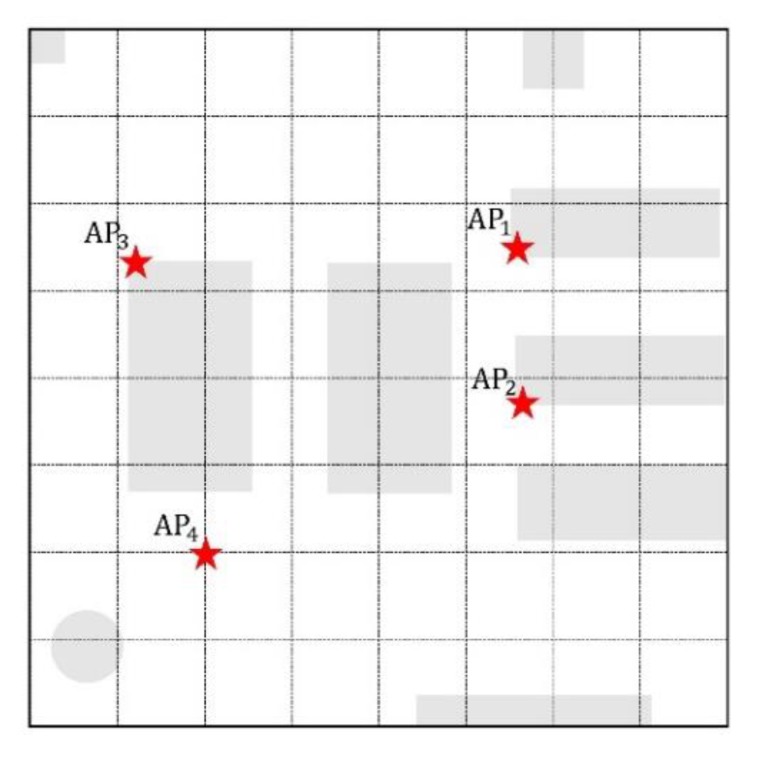
The plan of the experiments area.

In order to evaluate the performance of the proposed algorithm, experiments were conducted in a room which was used as the reading room in Tian Gong Building of Nanjing Tech University. The panel in Figure 10 shows the room of dimensions of 8 m × 8 m, where 4 iBeacon APs are laid. In Figure 9, the star symbols represent the position of the APs, the shadow symbols represent furniture in the room and the dot symbols represent the RSSI sampling points with the 1 m interval. Due to the indoor environment restrictions, the sampling intervals were not strictly 1 m. The RSSI was respectively collected with the rate of 1 s for about 100 s at each sampling point and with the rate of 0.5 s for 20 s at each testing point. An independent coordinate system was established in the room for convenience.

### 4.2. Comparison of Locating Accuracy

**Figure 11:**
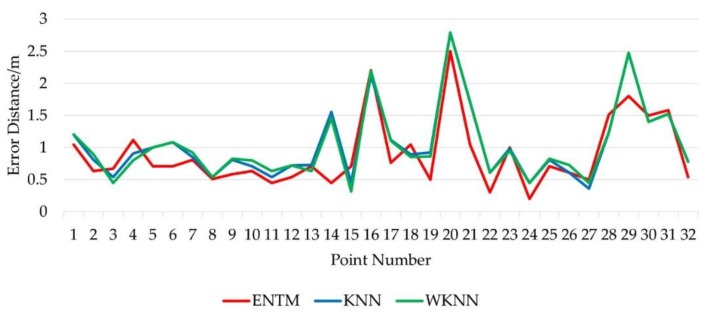
Comparison of the locating error of each testing point.

The fingerprint database was obtained by interpolating the original fingerprint sequence, and 32 testing points were randomly selected in the indoor environment. KNN, WKNN and ENTM algorithms were used to achieve static location. To compare the results of the experiment, the best locating results based on KNN and WKNN were respectively obtained by changing k. With the Euclidean distance between the locating result and the real position of the point taken as the accuracy standard, the best locating results were compared with each other. It shows that the error between them was the smallest when k = 7. Then the best locating results were compared with that of ENTM algorithm. The comparison of the locating accuracy of each point is shown in Figure 11.

It is shown in Figure 10 that the locating accuracy of the improved algorithm was superior to that of KNN and WKNN at points 5, 6, 14, 22 and 29. The maximum locating errors of the three algorithms were at the same point located at the edge of the room, where AP signal fluctuated greatly due to the nearby bookshelves. Further analysis indicated that the abnormal RPs were unavoidable to be selected in KNN and WKNN leading to lower locating accuracy, but the influence of the abnormal RPs may be filtered to a certain extent with the ENTM algorithm.

Table 2 is the comparison of the locating errors with the three algorithms, including the RMSE (Root of Mean Square Error) and the 80th percentile error. It may be seen that ENTM algorithm was 16.7% improved as compared with KNN and WKNN algorithms, and ENTM produced an 80th percentile error of 1.1 m which was 21.4% improved as compared with KNN and WKNN. The locating accuracy of ENTM was better than both KNN and WKNN.

**Figure 12:**
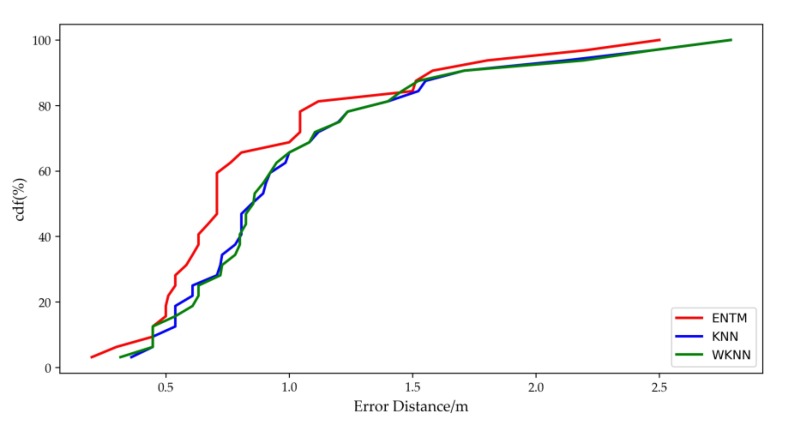
Comparison of locating error with three algorithms in terms of CDF (Cumulative Distribution Function).

Figure 12 shows the cumulative probability distribution of the locating error of the three algorithms. It can be seen in the figure that the probability of the locating error of less than 1.2 m with ENTM algorithm was 81.2%, but the probabilities with KNN and WKNN algorithms were both 71.9%.

## 5. Conclusions and Future Work

This paper proposes a BLE fingerprint indoor localization algorithm based on eight-neighborhood template matching. With the spatial resolution of BLE RSSI and the correlation of spatial RSSI fingerprint in the database analyzed, a method for the generation of eight-neighborhood template was proposed, which divided the indoor environment into four quadrants for each AP and chose the expected values of the RSSI difference between the center points and their eight-neighborhood in different quadrants as the generation parameters. RP was selected through template matching, which took the correlation of fingerprint data into account and may have filtered the influence of the abnormal RPs. The ENTM algorithm was tested through an experiment in a complex indoor environment. It showed that the locating error was 1.0 m, which was about 0.2 m less than that with KNN and WKNN algorithms.

There are a few researches on spatial fingerprint correlation in the field of fingerprint localization, and we believe that our work may inspire researchers to improve locating accuracy based on RSSI correlation. Since the acceptable localization results are illustrated with a relatively small room, we will expand the experimental area to the whole floor. The increase of the location area means that there would be more data in the fingerprint database, and the number of RPs with abnormal RSSI would increase. We plan to design a solution based on the techniques of data mining and machine learning to evaluate the continuity of fingerprint data [30], and to establish a fingerprint database with MARS (Multivariate Adaptive Regression Spline) [31] or CMARS (Continuous Multivariate Adaptive Regression Spline) [32] to improve the robustness of the locating system.

## Figures and Tables

**Table 1:** Relationship between bluetooth low energy (BLE) received signal strength indication (RSSI) and physical distance.

No.	d (m)	RSSI (dBm)	ΔRSSI (dBm)
1	0.5	−63.08	/
2	1.0	−66.88	3.80
3	1.5	−69.10	2.22
4	2.0	−70.68	1.58
5	2.5	−71.91	1.23
6	3.0	−72.91	1
7	3.5	−73.75	0.84
8	4.0	−74.48	0.73
9	4.5	−75.13	0.65
10	5.0	−75.71	0.58
11	5.5	−76.23	0.52
12	6.0	−76.71	0.48
13	6.5	−77.15	0.44
14	7.0	−77.55	0.40
15	7.5	−77.93	0.38
16	8.0	−78.27	0.34

**Table 2:** Locating error statistics.

Algorithm	RMSE (m)	Improved (%)	80th (m)	Improved (%)
K-nearest neighbor (KNN)	1.2	0	1.4	0
Weighted K-nearest neighbor (WKNN)	1.2	0	1.4	0
ENTM	1.0	16.7	1.1	21.4
